# Hepatitis B Virus Testing and Care among Pregnant Women Using Commercial Claims Data, United States, 2011–2014

**DOI:** 10.1155/2018/4107329

**Published:** 2018-04-01

**Authors:** Aaron M. Harris, Cheryl Isenhour, Sarah Schillie, Claudia Vellozzi

**Affiliations:** Division of Viral Hepatitis, Centers for Disease Control and Prevention, Atlanta, GA, USA

## Abstract

**Introduction:**

Pregnant women should receive hepatitis B virus (HBV) testing with hepatitis B surface antigen (HBsAg), but it is unclear whether HBV-infected pregnant women are linked to care.

**Methods:**

We analyzed MarketScan™ commercial insurance claims. We included pregnant women, aged 10–50 years, with 42 weeks of continuous enrollment before (predelivery) and 6 months after (postdelivery) the first delivery claim for each unique pregnancy between 1/1/2011 and 6/30/2014. We identified claims for HBsAg testing by CPT code and described the care continuum among pregnancies with an associated ICD-9 HBV diagnosis code by demographic and clinical characteristics, including HBV-directed care ([HBV DNA or hepatitis B e antigen] and ALT test codes) and antiviral treatment (claims for tenofovir, entecavir, lamivudine, adefovir, or telbivudine) pre- and postdelivery.

**Results:**

There were 870,888 unique pregnancies (819,752 women) included. Before delivery, 714,830 (82%) pregnancies had HBsAg test claims, but this proportion decreased with subsequent pregnancies (*p* < 0.0001): second (80%), third (71%), and fourth (61%). We identified 1,190 (0.14%) pregnancies with an associated HBV diagnosis code: most were among women aged ≥ 30 years (76%) residing in the Pacific (34%) or Middle Atlantic (18%) regions. Forty-two percent of pregnancies with an HBV diagnosis received HBV-directed care (42% predelivery and 39% postdelivery). Antiviral treatment was initiated before delivery in 128 (13%) of 975 pregnancies and postdelivery in 16 (1.6%) pregnancies.

**Conclusions:**

While most of these commercially insured pregnant women received predelivery HBV screening, we identified gaps in HBV testing and the HBV care continuum which highlight potential targets for public health interventions.

## 1. Introduction

Hepatitis B virus (HBV) infection remains a major public health issue [1]. In the United States, an estimated 847,000 persons are living with chronic hepatitis B virus (HBV) infection [2], with about 14,000 attributable deaths annually [3]. Approximately one in four persons living with chronic HBV infection will die prematurely from liver cirrhosis, hepatocellular carcinoma, or liver failure [4].

Serologic testing for hepatitis B surface antigen (HBsAg) is the primary way to identify persons with HBV infection. The Advisory Committee on Immunization Practices (ACIP) recommends that all pregnant women receive HBsAg testing during each pregnancy to identify infants needing postexposure prophylaxis [5]. There are approximately 25,000 infants born to HBsAg-positive pregnant women annually [6], and, without intervention, transmission can occur in up to 85% of exposed infants; among infected infants approximately 90% will become chronically HBV-infected [5, 7]. Infants born to HBsAg-positive women should receive hepatitis B vaccine and hepatitis B immunoglobulin (HBIG) within 12 hours of birth, followed by completion of the hepatitis B vaccine series and postvaccination serologic testing [5]. Postexposure prophylaxis is up to 95% effective in preventing HBV transmission among infants born to HBsAg-positive mothers [8, 9]. While prophylaxis has been successful in reducing maternal-to-child HBV transmission, there has been little support to address healthcare needs of mothers with HBV infection [10].

Prior studies have used claims data to study hepatitis B using International Classification of Diseases, Ninth Revision, Clinical Modification (ICD-9-CM) codes to identify persons with HBV who have liver cirrhosis [11] and antiviral treatment rates during pregnancy among women with HBV [12]. Claims data have also been used to assess the proportion of pregnant women receiving HBsAg testing [13]. We are not aware of previous studies that evaluated the complete HBV care continuum, from testing to linkage to care and treatment, among pregnant and postpartum women using claims data.

As a first step to address gaps in the HBV care continuum for pregnant women with HBV infection, we used commercial claims data to describe HBsAg testing during pregnancy and assess whether pregnant women with hepatitis B are linked to recommended care and antiviral treatment pre- and postdelivery.

## 2. Methods

### 2.1. Data Source and Study Population

We obtained demographic, enrollment, and insurance claims data from Truven Health's MarketScan Commercial Claims and Encounters insurance claims database, for women aged 10 to 50 years, from January 1, 2011, through June 30, 2014. This database includes claims for millions of persons with private insurance under the age of 65 years. A unique enrollee identification number allows linkage of medical claims with outpatient pharmacy claims data. This secondary analysis of deidentified insurance claims data did not require ethics approval.

To identify unique pregnancies during the study period, we searched medical claims for delivery-related diagnosis and procedure codes [12]. We defined the delivery index date for each pregnancy as the first service date on which a delivery-related code was documented. During the study period, women could have multiple unique pregnancies and associated delivery index dates, if all other study inclusion criteria were met. Specifically, for pregnancies to be included in the study, women were required to be continuously enrolled both 42 weeks before and 6 months following the delivery index date.

### 2.2. HBV Testing and Care Continuum Description and Statistical Analysis

To evaluate HBV testing, care, and treatment during pregnancy, we searched both inpatient and outpatient insurance claims for Current Procedural Terminology (CPT) codes, International Classification of Diseases, Ninth Revision, Clinical Modification (ICD-9-CM) procedure codes, and ICD-9-CM diagnosis codes associated with each cascade step (Table 1). We first calculated the proportion of all unique pregnancies with a claim for at least one HBsAg test during the 42-week period prior to the delivery index date and also used the Cochran–Armitage test for trend to examine differences in HBsAg testing rates with each subsequent pregnancy.

We then described the HBV care continuum among all pregnancies in which the mother was diagnosed with chronic HBV, defined as at least one chronic HBV ICD-9-CM diagnosis code (070.22, 070.23, 070.32, 070.33, or V02.61) documented on a claim prior to the delivery index date. Pregnancies with at least one HIV diagnosis code (795.71, V08, 042, or 079.53) documented on a claim prior to delivery were excluded because we sought to evaluate the HBV care continuum for HBV monoinfected women. We defined engagement in HBV-directed care as an alanine aminotransferase (ALT) test in conjunction with either an HBV DNA or hepatitis B e antigen (HBeAg) test, either before (up to 42 weeks before) or after (up to 6 months after) the delivery index date, and calculated the proportion of HBV-infected pregnancies in which the mother was engaged in care. Among a subset of pregnancies with pharmacy claims available for review, we calculated the proportion of HBV-infected pregnancies in which the mother initiated HBV treatment, defined as at least one claim for antiviral treatment (tenofovir, entecavir, lamivudine, adefovir, or telbivudine) either before or after the delivery index date.

We fit simple logistic regression models, with general estimating equations to account for women with multiple pregnancies, to evaluate the differences in characteristics between pregnancies in which women did or did not receive recommended HBV care. Analyses were completed using SAS, version 9.3 (Cary, North Carolina). Statistical tests were considered significant at *p* < 0.05.

## 3. Results

**Figure 1:**
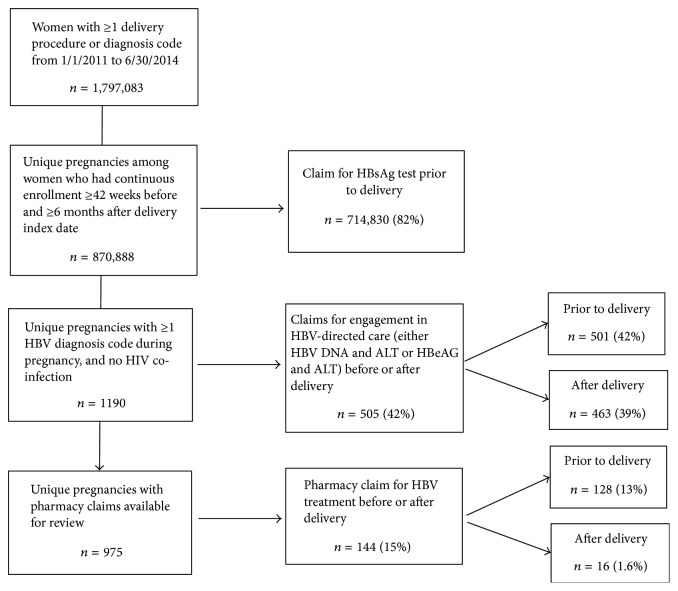
Flow chart of study criteria and cascade of hepatitis B testing, care, and treatment among pregnancies in MarketScan, 2011–2014.

There were 870,888 unique pregnancies (819,752 women) included (Figure 1). Of these, 714,830 (82%) had at least one HBsAg test claim prior to delivery. This proportion decreased with subsequent pregnancies (*p* < 0.0001, Table 2). HBsAg testing was more often performed in pregnancies occurring among women aged 20 to 39 years, residing in the North and South Central US, located in urban areas, and with a preferred provider organization (PPO) plan (Table 2). Lower HBsAg testing proportions were observed in pregnancies among women older than 40 years and those located in the Pacific census division.

We identified 1,190 of 870,888 (0.14%) pregnancies (1146 women) with an associated HBV diagnosis code, and six pregnancies that had an associated HIV diagnosis code were excluded. The proportion of pregnancies during which the mother was diagnosed with HBV increased with increasing age and was higher during pregnancies occurring in urban areas (Table 3). In addition, there were a higher proportion of pregnancies during which the mother was diagnosed with HBV in the Pacific and Middle Atlantic census divisions (Table 3).

Among the 1,190 pregnancies with an HBV diagnosis code, 505 received HBV-directed care. The proportion of pregnancies with HBV-directed care was similar predelivery and postdelivery (501 [42%] and 463 [39%], resp.). While there were geographic differences noted among pregnancies with HBV-directed care (with the greatest engagement in care among those in the West North Central and New England regions and the least engagement in care among those in the East North Central and Middle Atlantic regions), there were no significant differences by age groups, urbanicity, or insurance plan type of the women at the time of the pregnancy (Table 3).

Of the 1,190 pregnancies with an HBV diagnosis code, 975 (81.9%) had pharmacy claims available for review. Antiviral treatment was prescribed during 144 (15%) of these 975 pregnancies: predelivery in 128 (13%) and postdelivery in 16 (1.6%) (Table 4). There were no significant differences in maternal demographic characteristics for antiviral prescription status. Tenofovir (76%) was the most commonly prescribed antiviral medication followed by lamivudine (10%) and entecavir (9%); adefovir and telbivudine make up the remaining 5%.

## 4. Discussion

Using commercial claims data, we evaluated HBsAg testing practices and the HBV care continuum among commercially insured pregnant women. Most pregnancies (82%) had HBsAg test claim and 1,190 (0.14%) had an HBV diagnosis code. Our data demonstrated a gap in linkage to care among commercially insured pregnant women with HBV, as only 42% of pregnancies had claims suggesting HBV-directed care engagement. Furthermore, while an estimated 25% of persons with chronic HBV infection are treatment eligible, our study showed only 13% of pregnancies with an HBV diagnosis code were prescribed an antiviral prior to delivery, and 1.6% had an antiviral prescription postdelivery; however, we did not assess treatment eligibility (e.g., HBV DNA > 200,000 IU/mL) among these pregnancies.

Of a population of pregnant women with private health insurance, we showed many pregnancies (18%) without evidence of HBsAg test claim. CDC recommends that all pregnant women should be tested for HBsAg during an early prenatal visit (e.g., first trimester) in each pregnancy, even if they have been previously vaccinated or tested [5]. Testing provides documentation of the positive HBsAg test result and helps to ensure that infants will be identified for timely prophylaxis [5]. Previous data using MarketScan showed that 12% of insured pregnant women did not receive HBsAg testing [13], which differed from our data, possibly due to methodologic differences in amount of time in continuous enrollment (15 months in our study versus 12 months) and age groups included (10–50 years in our study versus 15–44 years). We also included more years of data (2011–2014 versus 2013-2014). Our analysis showed that women aged 20–39 years had higher testing proportions than women aged 40 years or older. This is consistent with the decreased proportion of women receiving HBsAg testing in subsequent pregnancies. While it is reassuring that a large proportion of pregnant women receive HBsAg testing, strategies are needed to get closer to 100% HBsAg testing.

Nationally and regardless of pregnancy status, few persons with HBV are linked to care and many public health efforts are underway to fill this gap [14, 15]. Even though a large number of pregnant women received HBsAg testing, many HBsAg-positive women were not linked to care [10]. Our data indicated that 58% of pregnancies with an associated HBV diagnosis code were not linked to appropriate HBV care either during pregnancy or postpartum. There were geographic differences for linkage to care; however, there were not significant differences for HBV linkage to care identified during pregnancy in urban versus nonurban areas. In addition to reducing maternal-to-child transmission, HBsAg testing during pregnancy provides an opportunity to link women to care. Linking HBsAg-positive pregnant women to HBV-directed care is the critical second step in the care continuum to identify those who may benefit from antiviral therapy which can delay HBV-associated liver complications and prevent maternal-to-child transmission.

In addition to prophylaxis for infants born to HBsAg-positive women, pregnant women with HBV may require antiviral treatment. The American Association for the Study of Liver Diseases (AASLD) guidelines suggest maternal antiviral therapy when the maternal HBV DNA is > 200,000 IU/mL [16]. Regarding antiviral treatment prescriptions, a prior analysis using commercial claims data showed a 13% antiviral prescription rate overall among pregnant women, which was significantly lower than that among nonpregnant women (20%) with HBV [12]. About 25% of persons with HBV are eligible for antiviral therapy [14]. Our data showed that 15% of pregnant women with an HBV diagnosis code were prescribed an antiviral overall and only 1.6% were prescribed an antiviral postdelivery. Tenofovir is first-line antiviral medication for treatment eligible pregnant women, although lamivudine or telbivudine has also been used as there is no evidence of adverse outcomes in infants born to mothers treated with these agents [16]. Although 11 pregnant women were treated with entecavir predelivery, entecavir is not recommended because the safety during pregnancy is not known. Our analysis showed that tenofovir was most commonly used, followed by lamivudine and entecavir. We did not include interferon in our analysis because it is contraindicated during pregnancy. Even though our analysis included claims data between 2011 and 2014, which is prior to the publication of updated AASLD clinical guidelines in 2016 [16], our analysis demonstrated significant linkage to care and treatment gaps among pregnant women with HBV.

Our analysis was subject to at least five limitations. First, we used a commercially insured claims database that does not include women without insurance and therefore does not represent the general US population. The HBV care continuum among under- or uninsured women and children still needs to be characterized to better target public health interventions. Second, there may have been misclassification related to claim codes used to identify pregnant women. For instance, if a claim was not submitted or reimbursed, it may not have been captured in the dataset, thereby leading to an underestimated sample. Third, there was potential for misclassification of HBsAg testing. We included a broad set of codes to include all potential HBV diagnoses; however, codes may have not equated to actual HBV infection as we were unable to confirm infection status with laboratory results or medical record review. Fourth, not all women had prescription claims data available for analysis, which may have led to an underestimated proportion of women receiving antiviral prescriptions. Fifth, we were not able to ascertain the proportion of pregnancies that would have been eligible for treatment, such as those with HBV DNA > 200,000 IU/mL, because we did not have laboratory data. In addition, since we defined care engagement as having one ALT, HBV DNA, or HBeAg, we were unable to assess continued follow-up care beyond initial care engagement. However, our results are consistent with other published data [12].

There have been several strategies identified that lead to increased testing and linkage to care for persons with HBV that may be applied to address gaps for pregnant women with HBV infection identified in this study. Patient education has shown success by facilitating increased HBV testing in hard to reach populations [15, 17, 18] and can be applied to pregnant women. In addition, provider education is important to ensure HBV testing is performed and correctly interpreted, and persons with positive results receive appropriate management [19], which can be applied to prenatal care clinics. Coordination of care through care managers or peer navigators has been shown to increase linkage to care [15, 20] and can be applied postdelivery to mothers identified with HBV. Utilization of health information technology may increase hepatitis B testing for pregnant women and prompt the provider to order appropriate HBV tests both pre- and postdelivery [21]. Further study is warranted to identify best practice strategies to increase linkage to care for pregnant women with HBV infection.

## 5. Conclusion

Commercial claims data can be used to evaluate the hepatitis B care continuum among commercially insured pregnant women. Our data showed that many pregnant women received predelivery hepatitis B testing; however testing should approach 100% among a commercially insured population. Furthermore we identified gaps in the hepatitis B care continuum among HBV-infected women which highlight potential targets for public health interventions to decrease HBV-associated morbidity and mortality among pregnant women and their infants.

## Figures and Tables

**Table 1:** Procedure^a^ and diagnosis codes^b^ used to identify hepatitis B diagnosis, testing, care, and treatment.

Test or condition	Code number	Code description
HBsAg test^a^	87340	Hepatitis B surface antigen
	80055	Obstetric panel
	80074	Acute hepatitis panel

HBV^b^	070.22	Chronic viral hepatitis B with hepatic coma without hepatitis delta
	070.23	Chronic viral hepatitis B with hepatic coma with hepatitis delta
	070.32	Chronic viral hepatitis B without mention of hepatic coma without mention of hepatitis delta
	070.33	Chronic viral hepatitis B without mention of hepatic coma with hepatitis delta
	V02.61	Hepatitis B carrier

ALT test^a^	80076	Hepatic Function Panel
	84460	Alanine Aminotransferase (ALT)

HBV DNA test^a^	87515	Hepatitis B virus detection by nucleic acid using direct probe technique
	87516	Hepatitis B Virus DNA, Qualitative, Real-Time PCR
	87517	Hepatitis B Virus DNA, Quantitative, Real-Time PCR

HBeAg test^a^	87350	Hepatitis Be Antigen

Newborn live birth^b^	V30–V39	Live born infants according to type of birth

HBIG administered^a^	J1573	Injection, hepatitis b immune globulin
	90371	Hepatitis B immune globulin

Hep B vaccine^a^	90739	Hepatitis B vaccine (HepB), adult dosage, 2 dose schedule, for intramuscular use
	90740	Hepatitis B vaccine, dialysis or immunosuppressed patient dosage (3 dose schedule), for intramuscular use
	90743	Hepatitis B vaccine, adolescent (2 dose schedule), for intramuscular use
	90744	Hepatitis B vaccine, pediatric/adolescent dosage (3 dose schedule), for intramuscular use
	90746	Hepatitis B vaccine, adult dosage (3 dose schedule), for intramuscular use
	90747	Hepatitis B vaccine, dialysis or immunosuppressed patient dosage (4 dose schedule), for intramuscular use

^a^Current Procedural Terminology or Healthcare Common Procedure Coding System. ^b^International Classification of Diseases, Ninth Edition, Clinical Modification.

**Table 2:** Hepatitis B screening among 870,888 pregnancies in commercial insurance claims data, 2011–2014.

		HBsAg^a^ tested		
	Total	*n*	Row%	*p* value^c^
Total	870,888^b^	714,830	82.08	
Pregnancy				**<0.001**
1st pregnancies	795,870	655,218	82.33	
2nd pregnancies	73,247	58,359	79.67	
3rd pregnancies	1,743	1,236	70.91	
4th pregnancies	28	17	60.71	
Age group				**<0.001**
19 or younger	39,067	28612	73.24	
20 to 29	356,474	300727	84.36	
30 to 39	438,844	363708	82.88	
40 or older	36,503	21783	59.67	
Census division^d^				**<0.001**
New England	40,403	32,767	81.10	
Middle Atlantic	102,312	80,774	78.95	
East North Central	161,920	130,865	80.82	
West North Central	45,137	38,475	85.24	
South Atlantic	153,566	128,662	83.78	
East South Central	52,726	45,441	86.18	
West South Central	100,916	89,101	88.29	
Mountain	56,611	47,470	83.85	
Pacific	134,756	103,260	76.63	
Other/unknown	22,541	18,015	79.92	
Urbanicity^e^				**<0.001**
Nonurban	106,490	85,273	80.08	
Urban	745,585	613,825	82.33	
Insurance plan type^f^				**<0.001**
Managed care^g^	207,179	159,265	76.87	
PPO or other^h^	637,739	533,989	83.73	

Boldface indicates statistical significance (*p* < 0.05); ^a^HBsAg = hepatitis B surface antigen. ^b^Among 819,752 women continuously enrolled 42 weeks before and 6 months after first delivery CPT or ICD-9-CM diagnosis code (Table 1).   ^c^Logistic regression models with general estimating equations to account for women with multiple pregnancies during the study period. ^d^New England = CT, MN, MA, NH, RI, VA; Middle Atlantic = NJ, NY, PA; East North Central = IN, IL, MI, OH, WI; West North Central = IA, KS, MN, MO, NB, ND, SD; South Atlantic = DC, DE, FL, GA, MD, NC, SC, VA, WV; East South Central = AL, KY, MS, TN; West South Central = AR, LA, OK, TX; Mountain: AZ, CO, ID, NM, MT, UT, NV, WY; Pacific = AK, CA, HI, OR, WA; and other/unknown = Puerto Rico and unknown state of residence. ^e^Urbanicity was determined by metropolitan statistical area (MSA); 15,732 HBsAg tested of 18,813 (83.6%) were missing urbanicity; ^f^21,576 HBsAg tested of 25,970 (83.1%) were missing insurance plan type. ^g^Health maintenance organizations (HMO), exclusive provider organizations (EPO), and point of Service (POS) plans. ^h^Preferred provider organizations (PPO), high deductible, and comprehensive plans.

**Table 3:** Characteristics of pregnancies by hepatitis B diagnosis and care engagement before and after delivery.

					Pregnancies with HBV diagnosis^a^ engaged^b^ before delivery^c^			Pregnancies with HBV diagnosis engaged after delivery^c^			Pregnancies with HBV diagnosis engaged EITHER before or after delivery^c^		
	Total pregnancies	Pregnancies with HBV diagnosis	row%	*p* value^c^	*n*	row%	*p* value^c^	*n*	row%	*p* value^c^	*n*	row%	*p* value^c^
Total	870888	1190	0.14		501	42.10		463	38.91		505	42.44	
Age group				**<0.001**			0.064			0.246			0.074
19 or younger	39067	6	0.02		0	0.00		0	0.00		0	0.00	
20 to 29	356473	282	0.08		126	44.68		109	38.65		126	44.68	
30 to 39	438839	797	0.18		346	43.41		329	41.28		350	43.91	
40 or older	36503	105	0.29		29	27.62		25	23.81		29	27.62	
Census division^e^				**<0.001**			**0.003**			**0.004**			**0.004**
New England	40403	48	0.12		28	58.33		27	56.25		28	58.33	
Middle Atlantic	102310	210	0.21		71	33.81		65	30.95		71	33.81	
East North Central	161920	153	0.09		34	22.22		33	21.57		37	24.18	
West North Central	45137	50	0.11		35	70.00		34	68.00		35	70.00	
South Atlantic	153565	127	0.08		56	44.09		52	40.94		56	44.09	
East South Central	52726	29	0.06		12	41.38		9	31.03		12	41.38	
West South Central	100914	120	0.12		52	43.33		44	36.67		52	43.33	
Mountain	56610	32	0.06		15	46.88		12	37.50		15	46.88	
Pacific	134756	401	0.30		190	47.38		181	45.14		191	47.63	
Other/unknown	22541	20	0.09		8	40.00		6	30.00		8	40.00	
Urbanicity^f^				**<0.001**			0.098			0.098			0.091
Nonurban	106490	33	0.03		9	27.27		8	24.24		9	27.27	
Urban	745579	1137	0.15		482	42.39		447	39.31		486	42.74	
Missing	18813	20	0.11		10	50.00		8	40.00		10	50.00	
Insurance plan type				**<0.001**			0.193			0.117			0.166
Managed care^g^	207178	384	0.19		171	44.53		161	41.93		173	45.05	
PPO or other^h^	637734	786	0.12		321	40.84		295	37.53		323	41.09	
Missing	25970	20	0.08		9	45.00		7	35.00		9	45.00	

Boldface indicates statistical significance (*p* < 0.05). ^a^At least 1 chronic HBV diagnosis code prior to delivery index date among pregnancies without HIV diagnosis. ^b^Defined as HBV DNA and ALT or HBeAG and ALT. ^c^Before (up to 42 weeks before) or after (up to 6 months after) the deliver index date. ^d^Logistic regression models with general estimating equations to account for women with multiple pregnancies during the study period. ^e^New England = CT, MN, MA, NH, RI, VA; Middle Atlantic = NJ, NY, PA; East North Central = IN, IL, MI, OH, WI; West North Central = IA, KS, MN, MO, NB, ND, SD; South Atlantic = DC, DE, FL, GA, MD, NC, SC, VA, WV; East South Central = AL, KY, MS, TN; West South Central = AR, LA, OK, TX; Mountain: AZ, CO, ID, NM, MT, UT, NV, WY; Pacific = AK, CA, HI, OR, WA; and other/unknown = Puerto Rico and unknown state of residence. ^f^Urbanicity was determined by metropolitan statistical area (MSA). ^g^Health maintenance organizations (HMO), exclusive provider organizations (EPO), and point of Service (POS) plans. ^h^Preferred provider organizations (PPO), high deductible, and comprehensive plans.

**Table 4:** Characteristics of 975 hepatitis B diagnosed pregnancies with pharmacy claims by antiviral treatment^a^.

		Pregnancies with HBV diagnosis treated before delivery^b^			Pregnancies with HBV diagnosis treated after delivery^b^			Pregnancies with HBV diagnosis treated EITHER before or after delivery^b^		
	Total	*n*	%	*p* value^c^	*n*	%	*p* value^c^	*n*	%	*p* value^c^
Total	975	128	13.13		16	1.64		144	14.77	
Age group				0.631			0.062			0.916
19 or younger	5	0	0.00		0	0.00		0	0.00	
20 to 29	232	33	14.22		1	0.43		34	14.66	
30 to 39	654	85	13.00		14	2.14		99	15.14	
40 or older	84	10	11.90		1	1.19		11	13.10	
Census division^d^				0.284			0.347			0.439
New England	36	7	19.44		1	2.78		8	22.22	
Middle Atlantic	143	27	18.88		1	0.70		28	19.58	
East North Central	127	10	7.87		1	0.79		11	8.66	
West North Central	44	5	11.36		1	2.27		6	13.64	
South Atlantic	105	13	12.38		3	2.86		17	15.18	
East South Central	27	4	14.81		0	0.00		3	11.11	
West South Central	112	16	14.29		1	0.89		13	11.61	
Mountain	27	3	11.11		0	0.00		2	7.41	
Pacific	346	42	12.14		8	2.31		50	14.45	
Other/unknown	8	1	12.50		0	0.00		1	12.50	
Urbanicity^e^				0.422			---			0.333
Non-urban	26	2	7.69		0	0.00		2	7.69	
Urban	939	125	13.31		16	1.70		141	15.02	
Missing	10	1	10.00		0	0.00		1	10.00	
Insurance Plan Type				0.405			0.162			0.566
Managed care^f^	329	49	14.89		8	2.43		57	17.33	
PPO or other^g^	629	78	12.40		8	1.27		86	13.67	
Missing	17	1	5.88		0	0.00		1	5.88	

Boldface indicates statistical significance (*p* < 0.05). ^a^Among 975 of 1190 HBV+ pregnancies where prescription drug claims were available for review. ^b^Before (up to 42 weeks before) or after (up to 6 months after) the deliver index date. ^c^Logistic regression models with general estimating equations to account for women with multiple pregnancies during the study period. ^d^New England = CT, MN, MA, NH, RI, VA; Middle Atlantic = NJ, NY, PA; East North Central = IN, IL, MI, OH, WI; West North Central = IA, KS, MN, MO, NB, ND, SD; South Atlantic = DC, DE, FL, GA, MD, NC, SC, VA, WV; East South Central = AL, KY, MS, TN; West South Central = AR, LA, OK, TX; Mountain: AZ, CO, ID, NM, MT, UT, NV, WY; Pacific = AK, CA, HI, OR, WA; and other/unknown = Puerto Rico and unknown state of residence. ^e^Urbanicity was determined by metropolitan statistical area (MSA). ^f^Health maintenance organizations (HMO), exclusive provider organizations (EPO), and point of service (POS) plans. ^g^Preferred provider organizations (PPO), high deductible, and comprehensive plans.

## Data Availability

MarketScan data is publically available.
